# A Randomized, Double-Blinded, Placebo-Controlled, Dose-Escalation Phase 1 Study of Aerosolized Pirfenidone Delivered via the PARI Investigational eFlow Nebulizer in Volunteers and Patients with Idiopathic Pulmonary Fibrosis

**DOI:** 10.1089/jamp.2018.1507

**Published:** 2020-01-30

**Authors:** Jun Keng Khoo, A. Bruce Montgomery, Kelly L. Otto, Mark Surber, Jessica Faggian, Jason D. Lickliter, Ian Glaspole

**Affiliations:** ^1^ Alfred Hospital, Monash University, Department of Medicine, Melbourne, Australia.; ^2^ Avalyn Pharma, Inc., Seattle, Washington.; ^3^ Nucleus Network, Melbourne, Australia.

**Keywords:** aerosol delivery, BAL, ELF concentrations, idiopathic pulmonary fibrosis, pirfenidone, vibrating plate nebulizer

## Abstract

***Background:*** This clinical trial evaluated the pharmacokinetics and safety/tolerability of inhaled pirfenidone solution in volunteers and patients with idiopathic pulmonary fibrosis (IPF).

***Methods:*** Forty-four adults in six cohorts consented to receive single doses of a 12.5 mg/mL pirfenidone solution or placebo to assess tolerability and pharmacokinetics. Cohorts 1, 2, and 3 (normal healthy volunteers [NHV]) (*n* = 6 active; *n* = 2 placebo in each cohort) received 25, 50, and 100 mg pirfenidone, respectively. Cohort 4 (NHV) (*n* = 6 all active) received 100 mg of pirfenidone and underwent bronchoalveolar lavage (BAL) to measure epithelial lining fluid (ELF) pirfenidone concentrations. Cohort 5 (prior or current smokers with greater than 20 pack-year use) (*n* = 6 active; *n* = 2 placebo) and Cohort 6 (IPF patients) (*n* = 6 all active) received 100 mg of pirfenidone. All treatments were administered with an Investigational eFlow^®^ Nebulizer System (PARI Pharma GmbH). Serial measures of urine and plasma pirfenidone were collected during the 24-hour postdose in all subjects.

***Results:*** Administration time ranged from 1.4 to 2 min/mL. No clinically relevant adverse effects on respiratory rate, spirometry, or oxygenation were observed. Drug-related adverse events were predominantly cough, *n* = 8/44 (one in IPF cohort), all mild, transient, and not dose limiting. Mean plasma pirfenidone Cmax levels in the 25, 50, 100 mg NHV, 100 mg smoker, and IPF cohorts were 202, 292, 802, 1370, 1016, and 1026 ng/mL, respectively. BAL cohort estimated ELF Cmax was 135.9 ± 54.5 μg/mL. In the BAL and IPF cohorts, 24-hour urine excretion of pirfenidone and metabolites data suggests similar alveolar deposition.

***Conclusions:*** Aerosol pirfenidone was well tolerated in normal volunteers, smokers, and IPF patients. High ELF concentrations were achieved in NHV with a 100 mg nebulizer dose. The 100 mg nebulizer dose averaged a 15-fold lower systemic pirfenidone exposure than reported with oral administration of the licensed oral dose.

## Introduction

Idiopathic pulmonary fibrosis (IPF) is a severe lung disorder characterized by progressive lung scarring, leading to increasingly disabling breathlessness and cough, and profound impacts on health-related quality of life. Death generally results within 2–5 years from the time of diagnosis, due to respiratory failure and/or comorbidities. It affects up to 200,000 Americans and 135,000 Europeans.^(1)^

Worldwide, two medications are currently approved for the treatment of IPF: oral nintedanib and oral pirfenidone. At the recommended dosing, both medications are associated with gastrointestinal side effects as well as liver enzyme elevation with nintedanib and photosensitivity and rash with pirfenidone.^(2,3)^ Adverse events (AEs) lead to dose reductions or discontinuations in many patients. In a 6-month study of compliance and persistence of both pirfenidone and nintedanib in 2331 newly prescribed patients, 23.8% patients in the pirfenidone cohort discontinued medication and 33.5% in the nintedanib cohort discontinued medication.^(4)^ Both drugs require lifetime therapy, and therefore, longer term discontinuation rates are likely higher.

Efficacy of both drugs is also not optimal, with each slowing the rate of disease progression as measured by serial forced vital capacity measures by about 50%.^(2,3)^

Aerosol administration of multiple classes of drugs, including bronchodilators, corticosteroids, and antibiotics, has been proven to improve both efficacy and safety by increasing delivery to lung tissue and decreasing systemic exposure.^(5)^ We investigated the safety and pharmacokinetics of a single administration of an aqueous formulation of pirfenidone delivered by a high-efficiency vibrating plate nebulizer to assess whether aerosol delivery of pirfenidone would be suitable for a longer term clinical trial that would assess safety and efficacy.

## Materials and Methods

This Phase 1 study was a randomized, double-blinded, placebo-controlled, dose-escalation study of sterile aqueous solution of either 12.5 mg/mL pirfenidone, 5 mmol/L citrate buffer, 150 mmol/L NaCl, and 0.75 mmol/L sodium saccharine and water, pH 6.0, or placebo containing 5 mmol/L citrate buffer, 150 mmol/L NaCl, 0.225 mmol/L sodium saccharine at pH 6.0 in volunteers and patients with IPF. All doses were delivered by the eFlow investigational vibrating mesh nebulizer (PARI, Gräfelfing, Germany), a continuous output device with no bias airflow and a holding chamber that increases delivery efficiency.^(6)^ Subjects were instructed to breathe normally as it was anticipated that controlled breathing patterns would not be maintained for the duration of administration times.

The study was conducted at Nucleus Networks in Melbourne, Australia, and the adjacent Alfred Hospital (bronchoscopy cohort). The study was approved by the hospital's Human Research Ethics Committee and was prospectively registered with the Australian New Zealand Clinical Trials Registry (ANZCTRN 12617001501336).

Cohorts 1, 2, and 3, all normal healthy volunteers (NHV) (*n* = 2 placebo; *n* = 6 active in each cohort), were conducted sequentially. Each received a single dose (25, 50, and 100 mg dose of aerosolized pirfenidone, respectively). In each of these cohorts, the first two (sentinel) subjects (one placebo/one active) were assessed for safety and tolerability before the remaining subjects were dosed.

Cohort 4 (NHV) (*n* = 6 active; no placebo) and Cohort 5 (prior or current smokers with at least a 20 pack-year history) (*n* = 2 placebo; *n* = 6 active) were administered the maximally tolerated dose (MTD) from Cohorts 1–3. Cohort 4 underwent bronchoalveolar lavage (BAL) to obtain epithelial lining fluid (ELF) pirfenidone levels after dosing and appropriate anesthesia. Three sequential 20 mL aliquots of normal saline were collected. Pooled aliquots after the first collection were used to quantify pirfenidone ELF levels. Measurements of BAL urea and serum were used to correct for dilution from the saline lavage. Cohort 5 received pretreatment with salbutamol immediately before inhalation of study drug.

Cohort 6 (IPF patients) (*n* = 6 active; no placebo) subjects were administered the MTD from Cohorts 1 to 3 after determination of safety and tolerability in Cohort 5 (prior or current smokers). Subjects with concomitant chronic obstructive pulmonary disease (COPD) or with smoking history or with current smoking status were pretreated with salbutamol immediately before inhalation of study drug.

Inclusion criteria for all cohorts were males or females, 18 ≤ age ≤55 years. Cohort 5 included only current or past smokers with >20 pack-year history of smoking. Cohort 6 included only patients with a diagnosis of IPF by American Thoracic Society/ERS (European Respiratory Society)/JRS (Japanese Respiratory Society)/ALAT (Latin America Thoracic Society) 2011 criteria, age <80 years, and for at least two subjects a greater than 20 pack-year smoking history.

Exclusion criteria were as follows: history of allergy or sensitivity to pirfenidone; use of oral pirfenidone in the 3 days before admission to Phase 1 facility; history of reactive airways disease (such as asthma or COPD), cystic fibrosis, or bronchiectasis (Cohorts 1–4 only); HIV-positive status, active hepatitis B or C; cigarette/e-cigarette smoking or use of other nicotine or tobacco containing products within 7 days before study drug administration (Cohorts 1–4 only); positive screen for drugs of abuse or alcohol; participation in a clinical study with administration of an investigational drug product within the previous 30 days, or five half-lives of the previously administered investigational product.

Other exclusion criteria were donation of blood or significant blood loss within the 8 weeks before admission to Phase 1 facility; donation of plasma within the week before admission to Phase 1 facility; any other condition that, in the view of the investigator, is likely to interfere with the study or put the subject at risk; and pregnancy or breast feeding.

For Cohorts 1–3 and 5, a randomization schedule was prepared by a statistician and a copy of the randomization code was kept on file at the investigational pharmacy. An unblinded pharmacist prepared study drug for dosing and labeled each dose in a blinded manner to ensure the investigator, clinic staff, and patient remained blinded.

As this was not a powered efficacy study, no formal sample size calculations were performed. The number of subjects proposed per cohort was considered sufficient for an exploratory study to assess the safety, tolerability, and pharmacokinetics of escalating doses of nebulized pirfenidone.

Pirfenidone plasma concentrations were measured at predose, 10 minutes, 30 minutes, and 1, 2, 4, 6, and 24 (±2) hours postcompletion of dosing in all cohorts (no 1-hour collection for Cohort 4 due to BAL procedure). To determine total delivered dose, all cohorts underwent 24-hour urine collection for pirfenidone and metabolite concentration measurements. To determine ELF concentrations, Cohort 4 subjects underwent BAL as soon as practical after dosing. The urea method was used to correct for BAL fluid dilution.^(7)^

Pirfenidone concentrations were determined by high-performance liquid chromatography–mass spectrometry/mass spectrometry method (MicroConstants, San Diego, CA). The following parameters were calculated for each dose: maximum plasma concentration (Cmax), time at which Cmax is attained (Tmax), and area under the curve from 0 to 24 hours (AUC 0–24). To estimate the Cmax concentrations in ELF of the BAL cohort immediately postdose, an ELF standard curve of clearance derived from serial BALs in a sheep model following inhalation of a pirfenidone formulation was used. This comparison assumes that a human would have a similar pirfenidone average ELF half-life to the sheep (7 minutes).^(8)^

Safety parameters evaluated included AEs, laboratory parameters (serial hematology, biochemistry, and urinalysis), change in spirometry after drug administration, and oximetry before and during drug administration.

All pharmacokinetic parameters were summarized using descriptive statistics and presented by time point and dose. All safety parameters were summarized using descriptive statistics and presented by dose.

## Results

### Tolerability

A total of 44 subjects were treated, 8 with placebo and 36 with aerosolized pirfenidone. Demographics of each cohort are reported in Table 1. All subjects in the smoker cohort had normal pulmonary function values. The IPF patients had mild to moderate disease with baseline FVC (forced vital capacity) values of 54%, 63%, 65%, 77%, 100%, and 124% predicted. All treatments were well tolerated with no clinically meaningful changes in oximetry, spirometry, vital signs, clinical chemistries, or hematology. No serious AEs were reported. Drug-related adverse effects are shown in Table 2; all were transient and mild, except for one moderate headache occurring several hours after drug administration and lasting for 2 days. Delivery time ranged from 1.4 to 2 minutes per mL of the pirfenidone solution or placebo.

**Table 1.** Demographic Characteristics

	All	Cohort 1	Cohort 2	Cohort 3	Cohort 4	Cohort 5	Cohort 6
	Placebo	Active	Active	Active	Active	Active	Active
	Subjects	25 mg	50 mg	100 mg	100 mg	100 mg	100 mg
	(*N* = 8)	(*N* = 6)	(*N* = 6)	(*N* = 6)	(*N* = 6)	(*N* = 6)	(*N* = 6)
Age (years)							
Mean (SD)	31.1 (12.3)	25.8 (6.2)	24.3 (2.4)	27.8 (4.1)	31.8 (5.9)	44.8 (5.8)	69.5 (4.3)
Median	25.0	24.5	24.5	28.5	32.0	45.5	68.5
Minimum, Maximum	20, 50	19, 37	20, 27	22, 32	25, 41	35, 51	64, 76
Gender							
Male	2 (25%)	2 (33%)	1 (17%)	3 (50%)	3 (50%)	4 (67%)	5 (83%)
Female	6 (75%)	4 (67%)	5 (83%)	3 (50%)	3 (50%)	2 (33%)	1 (17%)
FVC (mL)							
Mean (SD)	4.00 (1.04)	4.18 (1.08)	4.15 (0.86)	4.96 (1.42)	ND	3.91 (1.35)	2.93 (0.83)
Median	3.94	4.00	3.90	5.06	ND	3.49	2.69
Minimum, Maximum	2.77, 5.91	3.13, 6.16	3.20, 5.56	3.45, 6.49	ND	2.68, 6.44	2.24, 4.55

ND, not done; SD, standard deviation.

**Table 2.** Drug-Related Treatment-Emergent Adverse Events

	All	Cohort 1	Cohort 2	Cohort 3	Cohort 4	Cohort 5	Cohort 6
	Placebo	Active	Active	Active	Active	Active	Active
System organ class	Subjects	25 mg	50 mg	100 mg	100 mg	100 mg	100 mg
Preferred term	(*N* = 8)	(*N* = 6)	(*N* = 6)	(*N* = 6)	(*N* = 6)	(*N* = 6)	(*N* = 6)
Subjects with at least one drug-related TEAE	0	3 (50%)	2 (33%)	3 (50%)	1 (17%)	1 (17%)	2 (33%)
Respiratory, thoracic, and mediastinal disorders							
Cough	0	1 (17%)	1 (17%)	3 (50%)	1 (17%)	1 (17%)	1 (17%)
Possibly related	0	1 (100%)	0	0	0	0	1 (100%)
Probably related	0	0	1 (100%)	3 (100%)	1 (100%)	1 (100%)	0
Increased upper airway secretion	0	2 (33%)	0	0	0	0	0
Possibly related	0	2 (100%)	0	0	0	0	0
Probably related	0	0	0	0	0	0	0
Dysphonia	0	0	0	0	0	0	1 (17%)
Possibly related	0	0	0	0	0	0	1 (100%)
Probably related	0	0	0	0	0	0	0
Nervous system disorders							
Headache	0	0	1 (17%)	0	0	0	1 (17%)
Possibly related	0	0	1 (100%)	0	0	0	1 (100%)
Probably related	0	0	0	0	0	0	0
Dizziness							
Possibly related	0	0	1 (100%)	0	0	0	0
Probably related	0	0	0	0	0	0	1 (100%)

Severity was mild in all events with the exception of headache in IPF cohort that was moderate. This event occurred 5 hours after drug was administered and lasted for 2 days. All other related AEs were transient.

TEAE, treatment-emergent adverse event; IPF, idiopathic pulmonary fibrosis; AE, adverse event.

### Pirfenidone plasma pharmacokinetics

The pirfenidone plasma pharmacokinetics is presented in Table 3. As expected, pirfenidone was rapidly absorbed following administration; levels were low and below level of quantitation by 24 hours. The mean Cmax was correlated with dose. The volunteers with smoking history and IPF patients had a similar Cmax when compared with NHV. The average plasma half-life (t1/2) in the IPF patients was longer (3.87 vs. 2.01 hours), likely reflecting slower absorption from the lung rather than slower clearance. This notion is supported by the observation, as noted below; the systemic absorption measured over 24 hours in the IPF cohort was similar to other cohorts, as shown in Table 4.

**Table 3.** Plasma Pirfenidone Pharmacokinetic Parameters

Analyte	Cohort 1	Cohort 2	Cohort 3	Cohort 4	Cohort 5	Cohort 6
	Active	Active	Active	Active	Active	Active
	25 mg	50 mg	100 mg	100 mg	100 mg	100 mg
Parameter (unit)	(*N* = 6)	(*N* = 5)^a^	(*N* = 6)	(*N* = 6)	(*N* = 6)	(*N* = 6)
Pirfenidone (cont.)						
Cmax (ng/mL)						
Mean (SD)	202.3 (100.0)	292.2 (228.2)	802.6 (605.4)	1370.0 (769.7)	1016.0 (179.1)	1026.0 (117.7)
CV%	49.4	78.1	75.4	56.2	17.6	11.5
Median	209.0	276.0	693.5	1345.0	1001.0	995.5
Minimum, Maximum	70, 336	63, 630	52, 1900	306, 2490	824, 1310	894, 1230
Geometric Mean	178.4	215.0	540.9	1139.0	1003.4	1020.6
CV% Geometric Mean	63.7	118.5	185.2	85.6	17.3	11.1
t1/2 (hours)						
Mean (SD)	2.45 (0.35)	3.21 (0.75)	2.34 (0.54)	2.53 (0.70)	2.01 (0.43)	3.87 (1.15)
CV%	14.0	23.4	23.0	27.7	21.6	29.7
Median	2.32	3.28	2.10	2.38	1.95	4.21
Minimum, Maximum	2.15, 3.05	2.10, 4.15	1.84, 3.12	1.92, 3.56	1.48, 2.68	1.84, 5.12

^a^ One subject's dose was splashed out of device (unknown amount lost) so PK data unevaluable.

PK, pharmacokinetic, CV%, coefficient variation.

**Table 4.** Urine Carboxypirfenidone Ae0–24 (mg)

	Cohort 1	Cohort 2	Cohort 3	Cohort 4	Cohort 5	Cohort 6
	Active	Active	Active	Active	Active	Active
	25 mg	50 mg	100 mg	100 mg	100 mg	100 mg
	(*N* = 6)	(*N* = 5)^a^	(*N* = 6)	(*N* = 6)	(*N* = 6)	(*N* = 6)
Mean (SD)	10.4 (5.5)	11.2 (8.5)	49.0 (22.7)	40.3 (21.5)	46.7 (5.3)	42.2 (14.8)
CV%	53.1	75.9	46.2	53.4	11.4	35.0
Median	10.2	10.2	56.3	47.3	47.3	45.3
Minimum, Maximum	4.2, 19.2	2.3, 24.9	3.6, 64.0	14.0, 64.6	39.5, 55.1	18.8, 59.3
Geometric mean	9.2	8.6	36.4	34.0	46.5	39.6
CV% geometric mean	61.3	106.9	164.0	78.2	11.4	44.1

Ae0–24 urine excretion of four sequential 6-hour collections.

^a^ One subject's dose was splashed out of device (unknown amount lost) so PK data unevaluable.

### Pirfenidone urine pharmacokinetics

The carboxypirfenidone recovered in urine is shown in Table 4. Carboxypirfenidone represented at least 98% of total pirfenidone and metabolites in every subject's urine (data not shown). This indicates on average about 45% systemic absorption of the nebulizer dose. The predominate amount of pirfenidone was collected in the initial two 6-hour aliquots.

### Pirfenidone ELF levels

BAL was collected ∼45 minutes postdosing. Peak ELF pirfenidone concentrations from the pooled aliquot were obtained by correcting for dilution by the urea method and then extrapolating back to immediate postdose from the ELF clearance curve created from serial BALs in a sheep model of inhaled pirfenidone. The resulting pirfenidone peak ELF levels were 116, 14, 336, 120, 98, and 129 μg/mL, with mean ± standard deviation 135.4 ± 106.9 μg/mL. There was high variability in ELF concentrations, indicating the variability of aerosol administration. Two subjects' ELF concentrations were outliers; one low and one high, likely due to shallow breathing and deep breathing patterns, respectively.

## Discussion

The purposes of this Phase 1 study were to determine the pharmacokinetics, safety, and tolerability of nebulized pirfenidone in NHV, volunteers with an extensive smoking history, and patients with IPF.

A total of 44 subjects were enrolled: 30 healthy volunteers, 8 volunteers with extensive smoking history, and 6 IPF patients. The aerosol solution was well tolerated by all subjects. No serious AEs were reported. Drug-related AEs were mainly mild, intermittent cough seen in a minority of subjects. All subjects completed dosing in less than 15 minutes.

The 12.5 mg/mL pirfenidone concentration was chosen due to the limited solubility of pirfenidone. The 150 mM NaCl was added to prevent cough caused by solution lacking adequate permeable ions.^(9)^ Saccharin was added to mask a bitter taste and the solution was buffered to pH of 6.0.

To place context on the pirfenidone pharmacokinetics, the approved dose of oral pirfenidone is 801 mg three times per day. Following an 801 mg oral dose, systemic absorption is ∼85% (680 mg), and the peak plasma mean concentration is 7.9 μg/mL.^(10)^ A 100 mg nebulizer dose, with ∼45% systemic absorption (45 mg) as determined by 24-hour urine collection of pirfenidone and metabolites, leads to less than 1/15 the systemic exposure of the oral dose. Moreover, the peak plasma mean concentration is 1.7 μg/mL.

Peak ELF levels are of interest, as, in preclinical models showing efficacy, rapid clearance suggests the efficacy is related to peak ELF concentrations rather than ELF AUC. Assuming that free unbound pirfenidone is freely permeable across the alveolar capillary membrane, and that 50% of plasma pirfenidone is protein bound, one can estimate the ELF Cmax following an 801 mg oral dose to be 3.9 μg/mL (50% of 7.9 μg/mL). Comparing this with the 100 mg dose from the BAL cohort pooled aliquots, mean Cmax ± standard deviation (SD) in the ELF was 135.9 ± 108 μg/mL. This suggests that the ELF Cmax from a 100 mg inhaled dose would be on average 35-fold (range from 35- to 100-fold) higher than achieved with the approved oral dose.

The 24-hour urine carboxypirfenidone (the predominate form of urinary excretion) (mean ± SD) was 40.2 ± 20.5 mg and 42.2 ± 14 mg in the BAL and IPF cohorts, respectively. Peak plasma pirfenidone levels in these cohorts were 1.4 ± 0.7 and 1.0 ± 0.1 μg/mL, respectively. Because both cohorts absorbed a similar amount of drug, these data suggest that the IPF cohort (perhaps due to the decreased lung surface area from disease and slower absorption) achieved a higher ELF Cmax than the normal healthy volunteer cohort.

The Capacity 004 Phase 3 study of oral pirfenidone established that a 400 mg TID (three times daily) regimen had a better AE profile and about half the efficacy of 801 mg TID regimen.^(10,11)^ This study demonstrated that AEs were dose related, not idiosyncratic. Therefore, the lower systemic exposure, with inhalation of a 100 mg nebulizer dose (∼40–45 mg delivered systemically), may lead to a superior safety profile. In addition, a dose/response was demonstrated for the reduction in decline of FVC % predicted and progression-free survival in the CAPACITY 004 trial. Higher local concentrations delivered via aerosol administration may also lead to improved efficacy.

Supporting the hypothesis that improved efficacy with an aerosol dose is possible are three preclinical observations. The first is that the inhaled Cmax exceeds pirfenidone IC50 (∼25 μg/mL) in *in vitro* models and nears the observed EC50 (∼100 μg/mL) in animal models of bleomycin injury.^(12)^ As noted before, the ELF Cmax of the 801 mg oral dose is estimated at 3.9 μg/mL.

Second, preclinical models of IPF using bleomycin suggest that efficacy is correlated with Cmax rather than AUC.^(13)^ In one experiment, bleomycin efficacy in mice was seen with a once a week intratracheal aerosol dose even though the mouse pulmonary T1/2 of pirfenidone is <30 seconds, (personal communication Mark Surber).

Third, a recent study of aerosolized pirfenidone in a paraquat fibrosis model also showed that delivery of pirfenidone by inhalation achieved similar results to the oral route at substantially lower doses.^(14)^

A potential concern would be toxicity of high Cmax to the alveolar epithelial cells. Six-month aerosol pirfenidone exposures in a 6-month rat toxicology study with a plasma pirfenidone Cmax ∼2-fold higher than delivered to humans with a 100 mg nebulizer dose did not show any lung histology findings (S. Beck, pers. comm.).

This study supports the potential use of 25, 50, or 100 mg aerosol doses in future studies. If the target tissue is the alveolar epithelium, the Cmax in the ELF with the aerosol doses would be 8-, 17-, and 35-fold higher than that achieved with an 801 mg oral dose. With inhaled aerosol administration, lower drug levels would be achieved in the lung interstitial space, suggesting that any future study should consider use of the 100 mg arm as that is likely the highest practical dose.

This study did not address the issue of required frequency of dosing. As noted above, in a preclinical bleomycin model, once a week dosing led to efficacy. No clinical study of oral pirfenidone in IPF patients has tested dosing frequencies less than three times a day. However, because oral doses are given with meals, the time between the evening and morning dose is likely 12 or more hours, suggesting that twice-daily aerosol dosing should be considered in a future trial.

In conclusion, a 12.5 mg/mL pirfenidone aerosol solution was well tolerated in normal volunteers, volunteers with extensive history of smoking, and IPF patients. Pharmacokinetic data indicate higher lung levels after inhaled aerosol administration than that reported with administration of the approved oral dose. Further studies of aerosolized pirfenidone examining longer term safety and efficacy are warranted.

## Notes

Reviewed by:

Mark Utell

Steven Nathan

Rany Condos
